# Removal of Endocrine Disrupting Chemicals from Water: Adsorption of Bisphenol-A by Biobased Hydrophobic Functionalized Cellulose

**DOI:** 10.3390/ijerph15112419

**Published:** 2018-10-31

**Authors:** Antonio Tursi, Efthalia Chatzisymeon, Francesco Chidichimo, Amerigo Beneduci, Giuseppe Chidichimo

**Affiliations:** 1 Department of Chemistry and Chemical Technologies, University of Calabria, Via P. Bucci, Cubo 15D, 87036 Arcavacata di Rende (CS), Italy; amerigo.beneduci@unical.it (A.B.); giuseppe.chidichimo@unical.it (G.C.); 2 School of Engineering, Institute for Infrastructure and Environment, University of Edinburgh, The King’s Buildings, Edinburgh EH9 3JL, UK; E.Chatzisymeon@ed.ac.uk; 3 Department of Environment and Chemical Engineering, University of Calabria, Via P. Bucci, Cubo 41B, 87036 Arcavacata di Rende (CS), Italy; francesco.chidichimo@unical.it; 4 SIRiA S.r.l.-Servizi Integrati e Ricerche per l’Ambiente, c/o Department of Chemistry and Chemical Technologies, Spin-Off of the University of Calabria, Via P. Bucci, Cubo 15D, 87036 Arcavacata di Rende (CS), Italy

**Keywords:** endocrine disruptors, wastewater treatment, priority pollutants, surface modified cellulose, EDCs, Spanish broom

## Abstract

The aim of this study is to examine the efficiency of biobased Spanish broom (SB) surface modified cellulose fibers to remove bisphenol A (BPA), a well-known endocrine disruptor, from water. Spanish brooms are flowering plants, which are native and abundant to Mediterranean regions. The functionalized fibers (FF) were found to have the best adsorption efficiency at pH 5, due to the optimal hydrophobic interaction between the FF fiber and BPA. Adsorption kinetics of BPA was found to fit well a pseudo-second order reaction. Equilibrium isotherm data were fitted by Langmuir and Freundlich models. A very fast and simple regeneration method was developed and it was observed that adsorption capacity of the fibers was kept almost unchanged after 3 consecutive uses. Bottled water and synthetic wastewater were also tested to assess the efficiency of the process under more realistic water and wastewater treatment conditions. It was found that BPA removal was slightly decreased from 77% in ultrapure water to 64% in synthetic wastewater matrix, indicating that FF has a high selectivity toward BPA, even in the presence of other organic compounds. Overall, it was observed that SB-modified fibers can be a new promising green biotechnology for water purification.

## 1. Introduction

Endocrine disrupting chemicals (EDCs) are natural or synthetic compounds that have the ability to generate alterations in endocrine functions within the body and to interact with several classes of nuclear receptors and hormone receptors associated with endocrine and steroid hormones [1]. One of the most identified emerging EDCs is bisphenol A (BPA), a fundamental organic chemical used as a monomer in the production of polycarbonate plastics, epoxy resins and flame retardants [2]. The global production of BPA is about 8 million tons per year. This huge BPA production and processing volumes are responsible of the high presence of this substance in the environment [3]. Wastewater treatment plants, for example, inadvertently release BPA in effluents since they are not initially designed to treat such persistent and xenobiotic substances [4]. Huang et al. [5] found that the concentration of BPA is higher in industrial and commercial areas than in other regions, affecting water quality of rivers and lakes close to those areas. Moreover, recent studies by Yamamoto et al. [6] have shown that BPA can reach values up to 17.2 mg/L in landfill leachates, probably due to the presence of plastic debris. High BPA concentrations were also found in sewage treatment plant influents and in effluents of waste paper recycling plants (up to 370 μg/L) [7], in river water in Germany (up to 0.8 μg/L) [8], in wastewater in Ontario (up to 149 μg/L) [9], and in bottled water in France (0.07–4.21 μg/L) [10], to name but a few.

Drinking water treatment technologies are usually not able to remove the entire amount of BPA present in source waters [11], therefore there is a high risk of long-term exposure of humans to this compound. In this direction, numerous studies have been performed to investigate the correlation between BPA exposure and effects on health. These publications show the capability of this compound to increase risk for cancers, in particular ovarian, breast and prostate cancer [12,13], as well as various metabolic disorders such as obesity, endometrial hyperplasia, recurrent miscarriages and polycystic ovary syndrome [14,15]. Other different disorders associated with BPA exposure are cardiovascular disease, altered immune system activity, diabetes in adults, infertility and precocious puberty [16].

Several technologies, such as coagulation/flocculation/sedimentation/filtration [17,18], carbon nanomaterials (CNMs) (e.g., carbon nanotubes (CNTs) [19] and graphene oxides (GOs) [20]), membrane filtration [21,22], ultraviolet (UV) irradiation [23,24], biological processes [25,26], and advanced oxidation techniques [27,28,29,30,31] have been studied for BPA removal. Nevertheless, energy consumption, low process efficiency, production of toxic by-products, and addition of hazardous chemicals prevent the wider application of all these technologies at large-scale. Therefore, effective, more sustainable methods are sought to remove such contaminants from water and wastewater [32,33].

Physical adsorption is generally considered an effective method for the removal of these types of organic molecules in aqueous medium, provided that adsorbents have large accessible internal and/or external surfaces. In this regard, Nakanishi et al. [34] studied the adsorption characteristics of BPA on natural adsorbents produced from a variety of wood chips. Asada et al. [35] stressed that the porous carbon produced by bamboo could be an interesting adsorbent for the removal of BPA from aqueous solution. Yoon et al. [36] found out that adsorption efficiency of six types of activated charcoal powder (PAC), with respect to BPA, ranges from 31% to >99% with an activated carbon dosage of 5 and 15 mg/L. Also, Lazim et al. [37] observed that coir pith removed 72% of BPA reaching a maximum adsorption capacity of 4.308 mg/g, followed by durian peel (70%, 4.178 mg/g) and coconut shell (69%, 4.159 mg/g).

The development of an effective, simple, renewable and low-cost method to remove BPA from aqueous environment is therefore of great environmental importance and will safeguard public health. Based on these considerations, this work investigates the possibility to use hydrophobized cellulose fibers extracted from Spanish broom (SB) plants as a new biobased technology to remove BPA from water. Spanish brooms is a flowering plant, which is native and abundant to the Mediterranean regions in southern Europe, southwest Asia and northwest Africa. Spanish brooms cellulose fibers were successfully applied, by our research group, to remediate total petroleum hydrocarbons and heavy metals polluted water [38,39]. The interest for these fibers stemmed from the new techniques developed at the University of Calabria, which allows the extraction of the SB cellulose fibers through a fast and automated process, thus making the production costs extremely competitive. Cellulose fibers are extracted with a typical length ranging from 5 to 50 cm, and diameters of the order of about 10 μm. These fibers are suitable to be applied in filtering processes, since they have a very high specific surface area. The surface of the fibers was chemically modified in order to increase its hydrophobicity, using the reactivity of the cellulose hydroxyl groups with the 4,4’-diphenylmethane diisocyanate (MDI) [38]. One of the isocyanate groups of this molecule is bound on the cellulose surface through a urethane link, while the other one is transformed in an amine group by water washing. In this way the fiber surface is coated by a chemical group which is very similar to BPA, and thus an expected high chemical affinity for this molecule is achieved. As previously reported [38], the SB fibers functionalization was carried out in a home-made reactor, by a direct nebulization of MDI on the surface of the fibers. This procedure avoids the use of solvents, reducing the chemical impact and the cost of the process.

In this study, batch experiments were performed in order to assess the remediation capacity of the modified SB fibers with respect to BPA polluted water. Kinetics and thermodynamics aspects of the adsorption process were studied. A method to regenerate the used fibers was developed and the removal efficiency of the regenerated fibers was studied.

## 2. Materials and Methods

### 2.1. Materials

All chemicals used in this study were of analytical grade and without further modification. Bisphenol A (CAS No: 80-05-7) was purchased from Sigma-Aldrich (Haverhill, Suffolk, UK); MDI (4,4’ diphenylmethane diisocyanate) was provided by The Dow Chemical Company (Pisticci Scalo, MT, Italy); acetone water free was purchased from Sigma-Aldrich.

The fibers of the vegetable, which grows naturally in the Mediterranean area, were obtained by using a pulping process in a 5% *w*/*w* NaOH solution. The vegetable was macerated for 20 min at a temperature of 80 °C. After this treatment the cellulose fiber could be easily separated from the inner woody skeleton of the SB bushes. Lignin impurities were removed by further washing the cellulose fibers with a 5% *w*/*w* NaOH solution at a temperature of 80 °C [40]. 

Experiments were performed by spiking appropriate amounts of BPA into various water matrices, such as ultrapure and bottled (with TOC = 4.2 mg/L; COD = 10 mg/L; pH = 7.2) water as well as synthetic wastewater. Synthetic wastewater (with TOC = 160.1 mg/L; COD = 367 mg/L; pH = 7.7) was prepared by dissolving the following components in one liter of distilled water: peptone, 160 mg; meat extract, 110 mg; urea, 30 mg; anhydrous dipotassium hydrogen phosphate (K_2_HPO_4_), 28 mg; sodium chloride (NaCl), 7 mg; calcium chloride dehydrate (CaCl_2_⋅2H_2_O), 4 mg; magnesium sulphate heptahydrate (Mg_2_SO_4_⋅7H_2_O), 2 mg. Total organic carbon (TOC) was determined by measuring the organic concentration by a TOC analyzer (Shimadzu TOC-VCPH). Chemical oxygen demand (COD) was determined colorimetrically by the dichromate method. Commercially available digestion solution containing potassium dichromate, sulfuric acid and mercuric sulfate (Palintest, Camlab, UK) was used. 

Biochemical Oxygen Demand (BOD_5_), total nitrogen (N) and total phosphorous (P) were determined according to the following standard methods: APAT IRSA CNR 5120 and 4060, respectively [41].

The measured BOD_5_/N/P ratios for synthetic wastewater are 100/22.7/2.3 which are close to typical values found for raw municipal wastewater [42]

### 2.2. Cellulose Fiber Hydrophobization

Hydrophobization of SB fibers was performed through a novel solvent-free technology based on a home-made steel reactor that keeps the fibers under vortex stirring by rotating blades, which were set at the bottom of the reactor. During this phase, the MDI reactant is spread onto the surface of the fibers in the form of micrometer-sized droplets, by two nebulizers, following the procedure described in Tursi et al. [38]. Both raw (RF) and functionalized (FF) fibers were chemically characterized by fast Fourier transform infrared spectroscopy (FT-IR) [38], confirming the presence of urethane bond in the FF fibers.

### 2.3. Cellulose Fiber Surface Characterization

Further support of the functionalization process comes from the Boehm titration analysis, which was used to measure the content of the acidic and basic functional groups on the fiber surface. This method measures the amount of NaOH, Na_2_CO_3_, and NaHCO_3_ neutralized by the acid groups present on the surface of a material, corresponding to weak, moderate and strong acid fractions, respectively. Hydrogen chloride was instead used to measure the basic functional groups content [43].

**Scheme 1:**
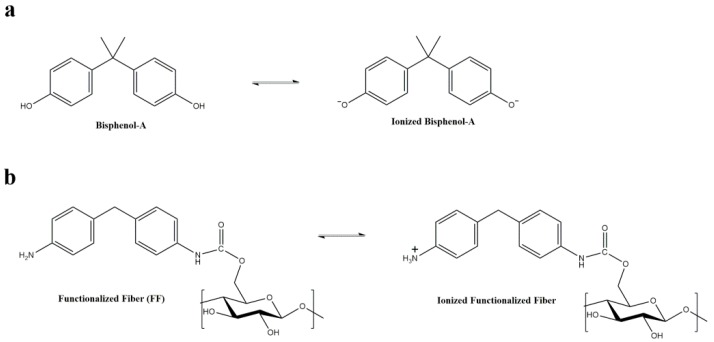
(**a**) Bisphenol-A (unionized and ionized forms), and (**b**) Functionalized cellulose fiber (unionized and ionized forms).

The weak, moderate and strong acid fractions of the FF are (0.018 ± 0.002) mmol/g, (0.035 ± 0.004) mmol/g, (0.005 ± 0.001) mmol/g, respectively, while the basic groups content is (0.814 ± 0.003) mmol/g, due to the grafting of ammine groups on the cellulose fiber surface (Scheme 1).

On the other hand, functionalization of the fiber does not significantly change the specific surface area (SSA) of the fiber as determined by the Brunauer–Emmett–Teller (BET-SSA) analysis of the raw (RF) and functionalized (FF) fibers which are 1.3 ± 0.4 m^2^/g and 1.4 ± 0.4 m^2^/g, respectively. The BET-SSA was measured with an Autosorb IQ Chemi TCD instrument (Quantachrome Instruments, Boynton Beach, FL, USA), utilizing adsorption–desorption N_2_ isotherms at 77 K. The cellulosic fiber extracted from the Spanish broom has a very large specific surface area compared to other natural fibers such as hemp and flax whose BET-SSA ranges from 0.31 to 0.88 m^2^/g [44].

### 2.4. BPA Analytical Determination

Bisphenol A concentration in filtrate samples was measured by means of a high-performance liquid chromatography (HPLC) system (S200 Pump, S225 Autosampler, Perkin Elmer, Beaconsfield, UK) coupled with a diode array detector (S200 EP, Perkin Elmer Beaconsfield, UK). Samples were separated by reverse phase (RP) chromatography using a C18 Luna (Phenomenex) column (5u, 250 × 4.6 mm). The HPLC method was obtained by Davididou et al. [27]. The mobile phase was a mixture of water/acetonitrile (35/65, *v*/*v*) at a flow rate of 1 mL/min using UV wavelength at 225 nm. The solvents were eluted isocratically and the injection volume was kept at 40 μL.

### 2.5. Adsorption Tests

The adsorption capacity of the SB functionalized fibers was studied by carrying out several batch experimental runs. In each experiment, a certain amount of fiber ranging from 10 to 20 g/L was added to the polluted water, which was obtained by spiking BPA in distilled water at an initial concentration ranging from 10 mg/L to 30 mg/L. Experimental temperature was 20 °C.

The initial pH of the reactant mixture was varied in order to study the effect of this parameter on the adsorption capacity, by using appropriate amounts of NaOH or HCl solutions.

Experiments were also performed with bottled water and synthetic wastewater. The system was maintained under magnetic stirring for the whole duration of the experiment. Bisphenol A concentration was monitored as a function of time (zero time was defined as the time that fibers were added in the reactant mixture) and samples were withdrawn at specific time intervals. 

The adsorption capacity, i.e., the amount of adsorbed BPA per unit mass of adsorbent (*q*_t_), was defined as:

(1) $$q_{t}=\frac{\left(C_{0}-C_{t}\right)V}{W}$$

where *C*_0_ is the initial BPA concentration (mg/L), *C_t_* is the one observed at time *t*, *V* is the solution volume (L) and *W* is the adsorbent mass (g). Once the equilibrium condition of the adsorption process is reached, *C_t_* = *C_e_* (equilibrium concentration) and *q_t_* = *q_e_* (equilibrium adsorption capacity).

Alternatively, data were reported in terms of removal efficiency (*RE*%), defined as the following Equation (2):

(2) $$RE\%=\frac{\left(C_{0}-C_{t}\right)}{C_{0}}100$$

## 3. Results and Discussion

### 3.1. Effect of pH 

**Figure 1:**
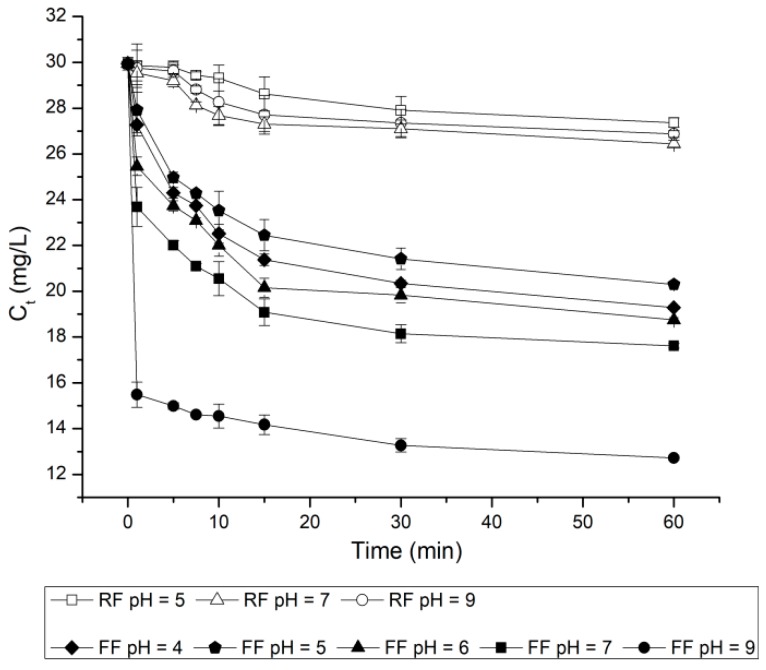
Effect of pH on the removal of Bisphenol A (BPA) by raw (RF) and functionalized (FF) fiber. Experimental conditions: fiber loading = 1 g; water volume = 100 mL; *C*_0_ = 30 mg/L; water matrix = UP.

The effect of pH on the adsorption efficiency of RF and FF was studied. Experiments were conducted at different pH values (4, 5, 6, 7, 9) in the presence of 30 mg/L of BPA and 1 g of fiber in 100 mL of water. Figure 1 shows BPA concentration as a function of contact time between the BPA and the fibers. As a preamble, three important observations can be made: (a) concentration of BPA decreases with time and tends to reach a plateau after about 15 min, due to the limited number of active sorption sites on the fiber; (b) BPA adsorption onto FF changes as a function of pH, with the highest removal (50%) taking place at pH 5; (c) removal efficiency of FF is significantly higher than that of the RF (only about 6% BPA removal), at all pH values assayed. 

The variation of adsorption capacity with pH can be qualitatively explained by taking into consideration the acid/base equilibrium of the interacting species (Scheme 1). The pH of the solution determines the ionization state of both the adsorbent (FF) and adsorbed (BPA) functional groups and thereby their hydrophobicity, thus governing the adsorbent-adsorbed interactions. The BPA molecule has a pK_a_ = 9.6 [2], which means that the fraction between the ionized (BPA^2−^) and unionized (BPA) forms increases with pH, reaching a considerable amount only at pH 9, at which roughly 1/4 molecules of BPA are ionized (at pH 9 it is about 0.25, at pH 7 is 0.0025 and at pH 5 is 0.000025). Therefore, at higher pH values the BPA solubility in water increases, i.e., it becomes more hydrophilic, thereby its tendency to be adsorbed onto hydrophobic surfaces decreases. 

In contrast, the effect of altering the pH on the amine function (aniline like) of the FF is the opposite: since the pK_b_ of aniline is 9.6, about 70% of amine residues are in the ionized (protonated) form at pH 4, while at pH values above 5 only a small fraction of the amine terminal groups is protonated. In other words, the hydrophobicity of the absorbing fiber surface decreases abruptly when pH decreases from 5 to 4. Moreover, there is no doubt that any anion/cation binding interaction plays an important role, since, when BPA is in the anion form at high pH, the surface of the fiber is neutral and, vice versa, when the fiber surface is in the cation form at low pH, BPA is neutral.

**Figure 2:**
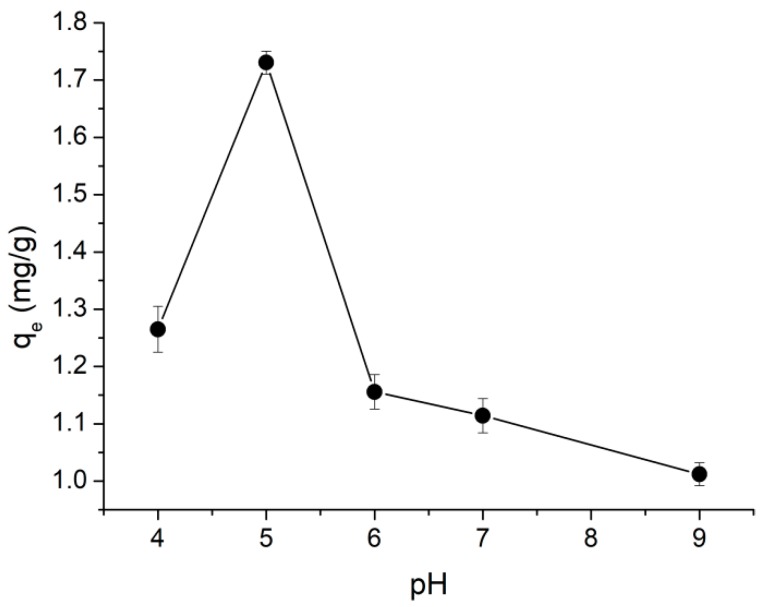
BPA equilibrium adsorption capacity *q_e_* as a function of pH. Experimental conditions: fiber loading = 1 g; water volume = 100 mL; *C*_0_ = 30 mg/L; water matrix = UP.

The data reported in Figure 1 shows qualitatively that the hydrophobic interaction between the functional group of the cellulose and the BPA, as well as the hydrophilic interactions of the ionic forms of both MDI amine residue and BPA molecules with water molecules playing a role in the sorption efficiency of FF fibers. Data shown in Figure 1 can be better discussed when observing Figure 2, where the plot of equilibrium adsorption capacity (*q_e_*) versus pH is presented. The maximum in *q_e_* around pH 5 is due to the optimal hydrophobic interaction between the FF fiber and BPA. At lower pH values, FF fibers gradually lose their hydrophobicity, which also happens to BPA at pH above 5, thus lowering the adsorption efficiency of the fibers. 

### 3.2. Regeneration and Reuse of Fiber

**Figure 3:**
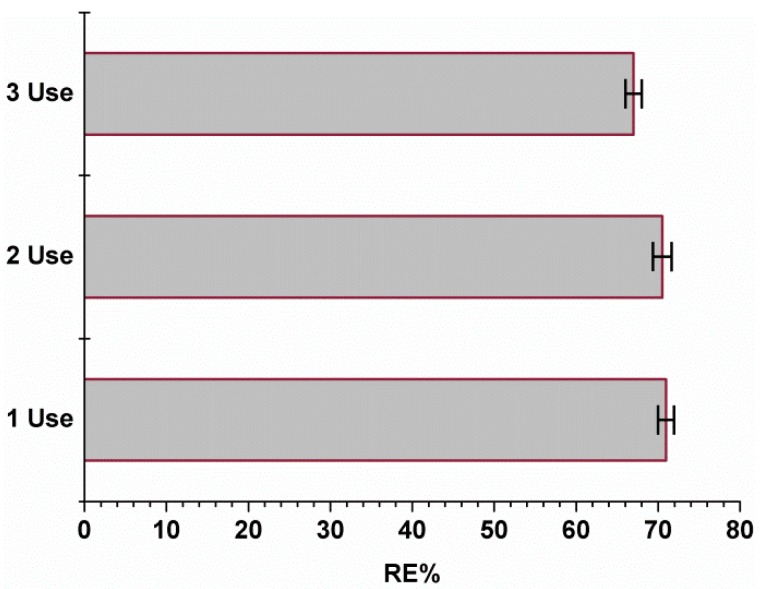
Bisphenol A removal efficiency, RE%, during regeneration and reuse of FF. Experimental conditions: fiber loading = 1 g; water volume = 50 mL; *C*_0_ = 15 mg/L; pH = 5; contact time = 60 min.

Recycling of the adsorbent material reduces the operational costs and the environmental footprint of the treatment process. Therefore, regeneration studies are important to assess the potential of applying this biobased treatment technology at large-scale. Regeneration of the fibers were performed with the aim to clean the adsorbent sites and use them again for BPA removal. The regeneration procedure was based on the ionic hydrophilic form that BPA takes when the pH increases, thus allowing pollutant desorption. For this reason, the used FF was washed for 2 min with a 0.1 M NaOH solution and then further washed with water to remove any possible NaOH residual on the fiber surface. After this very simple and quick treatment, the FF was used again for BPA removal, and the results are shown in Figure 3. It can be seen that the regenerated FF exhibits satisfactory reusability, since its adsorption capacity is very close to that of the original FF, which accounts for about 70% of BPA removal even after three consecutive regeneration cycles. 

### 3.3. Effect of Water Matrix

**Figure 4:**
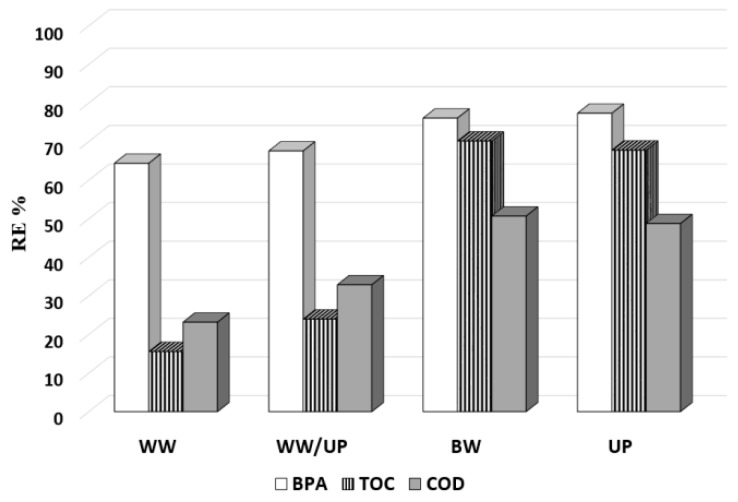
Removal efficiency, RE%, in various water matrices. Experimental conditions: fiber loading = 1 g; water volume = 50 mL; *C*_0_ = 15 mg/L; pH = 5; contact time = 60 min. The errors in the calculated removal efficiency are 1% for BPA, 2% for TOC and 4% for COD.

Further experiments were conducted in water matrices like ultrapure water (UP), bottled water (BW), synthetic wastewater (WW) and diluted WW in UP at a 50/50 ratio (WW/UP) in order to resemble more realistic water and wastewater treatment conditions. All water matrices were spiked with an initial BPA concentration of 15 mg/L and treated by 1 g of FF at a pH = 5 in a volume of 50 mL. Results are shown in Figure 4, where it can be observed that higher BPA removal (values shown in brackets) is achieved for experiments conducted in UP (77 ± 1%) followed by BW (76 ± 1%), WW/UP (67 ± 1%) and WW (64 ± 1%). This slight reduction in the RE% of BPA can be explained by the presence of other organic compounds such as urea, and those contained in peptone and meat extract in the WW/UP and WW, which interfere, to a lesser extent, with the adsorption of BPA, since they might compete with BPA for the active sites of the adsorbent. This is in agreement with the low COD and TOC removal efficiencies, which indicate that the FF does not have any specific preference for binding the organic components of WW other than BPA. In contrast, the fiber has a high selectivity toward BPA whose adsorption remains above about 64%, independently from the nature of the aqueous matrix in which it is dissolved.

These data highlight further the high affinity of FF surface for BPA, which is due to the functional group added, having a chemical structure very similar to that of the BPA molecules (Scheme 1). On the other hand, the low affinity of the FF surface for the other organic components of the WW is mainly due to their polar nature (amino acids, urea, etc.) having high affinity for water. 

### 3.4. Adsorption Kinetics of BPA

**Figure 5:**
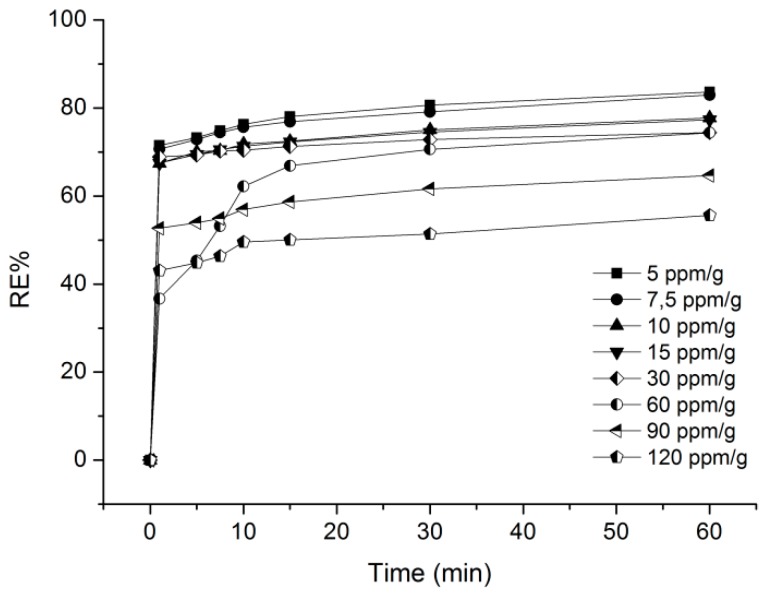
Process efficiency (%) in terms of BPA removal at various *C*_0_/*W* (i.e., initial BPA concentration/fiber mass) ratios. Experimental conditions: water volume = 50 mL; pH = 5. The errors in the calculated removal efficiency are in the range from 0.11 to 1.17%.

Several experimental runs were performed in order to investigate the kinetics of the process. In Figure 5, process efficiency in terms of BPA removal is shown. It can be observed that in the presence of 5–30 ppm BPA/g of FF, BPA removal is at 70 ± 1% after 5 min of treatment and increases up to about 79 ± 1% after 60 min. At higher C_0_/W ratios of 60, 90 and 120 ppm/g removal efficiency of BPA is substantially lower at 74 ± 1%, 64 ± 1% and 55 ± 1% after 60 min of treatment. 

**Figure 6:**
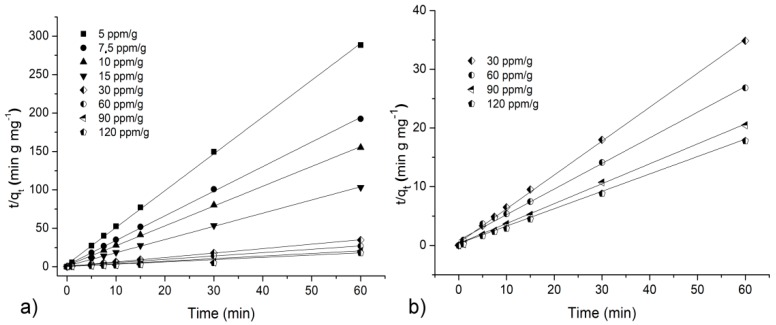
(**a**) Fitting of the experimental data by the pseudo-second order kinetics model. Experimental conditions: water volume = 50 mL; pH = 5. (**b**) Magnification of the data from 30 ppm/g to 120 ppm/g for a better visibility of the fitting. The errors in the t/*q_t_* evaluations are in the range from 0.09 to 0.72%.

Figure 6 shows that BPA adsorption kinetics were fitted very well by a pseudo-second order model, with excellent *R*^2^ values. The rate of change of the adsorption capacity is given by Equation (3) [45]:

(3) $$\frac{dq_{t}}{dt}=k_{2}{\left(q_{e}-q_{t}\right)}^{2}$$

where *k*_2_ and *q_e_* are the pseudo-second order rate constant (g/mg min) and the equilibrium absorption capacity (mg/g), respectively. Equation (4), obtained after integration and linearization of Equation (3), was used to estimate the parameters *k*_2_ and *q_e_* by fitting the *t*/*q_t_* ratio as a function of time (Table 1):

(4) $$\frac{t}{q_{t}}=\frac{1}{K_{2}q_{e}^{2}}+\frac{1}{q_{e}}t$$

The equilibrium adsorption capacity significantly increases with the *C*_0_/*W* ratio while k_2_ decreases in agreement with the hypothesis that the fiber surface has a high degree of chemical functionalization, i.e., a high number of adsorption sites.

### 3.5. Adsorption Isotherm

**Figure 7:**
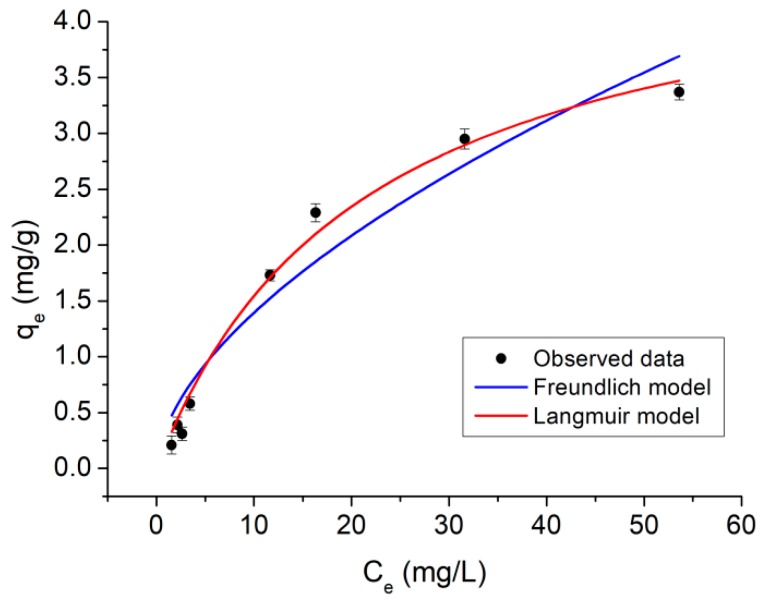
Bisphenol A adsorption isotherm at 20 °C onto biobased FF (black symbols) and fitting with the Langmuir (red line) and Freundlich (blue line) models.

The adsorption capacity of the FF in terms of BPA removal has been assessed by plotting the adsorption isotherm data (*q_e_* vs. *C_e_*), which has been fitted with Langmuir and Freundlich models (Figure 7).

As can be seen, the equilibrium adsorption capacity (*q_e_*) increases nonlinearly with the equilibrium concentration of BPA (*C_e_*) and almost reaches a plateau at *C_e_* ˃ 30 mg/L. 

The Langmuir model, which assumes that the surface of the adsorbing material is covered by a monolayer of the adsorbate, that the adsorption sites are all equivalent and that the solute particles adsorbed do not interact with each other [46], is expressed by Equation (5):

(5) $$q_{e}=\frac{q_{m}K_{L}C_{e}}{1+K_{L}C_{e}}$$

where *K_L_* is the Langmuir adsorption constant and *q_m_* is the maximum adsorption capacity of the FF in terms of BPA removal. Figure 7 shows an excellent agreement between the experimental and calculated values, indicating the goodness (*R*^2^ = 0.99) of the Langmuir model with estimated *K_L_* = (0.046 ± 0.008) L/g and *q_m_* = (4.86 ± 0.3) mg/g.

Worse results have been obtained by modeling the experimental data with the Freundlich model, according to Equation (6):

(6) qe=KFCe1n

where *K_F_* is the Freundlich adsorption constant, related to the surface heterogeneity. Figure 7 shows that the fitting curve (blue line) obtained with the Freundlich model (*R*^2^ = 0.935), to which correspond the following fitting parameters: *K_F_* = (0.4 ± 0.1) L*^n^*/g mg^(*n*−1)^ and *n* = (1.72 ± 0.23).

Natural materials, whose adsorbing capacities have been improved through intensive treatments with high environmental impact (e.g., carbonization), have higher adsorption capacities than those materials treated with eco-friendly processes. Among these last adsorbents (Coir pitch, Coconut shell, Durian peel), the SB FF has the highest adsorption capacity, shows the fastest adsorption kinetics, and works in a relatively wide pH range (4–6) (Table 2). 

## 4. Conclusions

This work reports the first application of biobased surface functionalized Spanish broom cellulose fibers to remove BPA, an endocrine disrupting chemical and priority pollutant, from water. It was found that pH significantly affects adsorption capacity with the highest adsorption rates being observed at pH 5. The functionalized cellulose fiber exhibited very good adsorption properties for BPA with 70% of the pollutant being adsorbed after 5 min of contact time. It was observed that the adsorbent system can be easily regenerated by a simple and fast washing process which allows it to recover its adsorption capacity by almost 100 % of the original one. These results indicate the potential for practical application of this technology, due to the high availability of cellulose coming from Spanish broom plants and the easily scalable process for cellulose functionalization. Other advantages of the Spanish broom fiber are: (1) its very easy extraction process [40], which gives a fiber with a high percentage of cellulose (>92%) and a relatively low lignin content (−3%) [49]; (2) its huge mechanical properties, with a tenacity of −36 cN/tex and a strain at break of −6%, which are high compared to, for instance, flax fibers [49].

## Figures and Tables

**Table 1:** Pseudo-second order kinetic data for experimental runs shown in Figure 5 and Figure 6.

*C*_0_/*W* (ppm/g)	*k*_2_^1^ (g mg^−1^ min^−1^)	*q_e_*^1^ (mg g^−1^)	*R* ^2^
5	7.175 (1.140)	0.21 (0.04)	0.99935
7.5	4.629 (0.885)	0.31 (0.03)	0.99913
10	4.311 (0.590)	0.39 (0.02)	0.99940
15	2.937 (0.393)	0.58 (0.02)	0.99940
30	0.757 (0.155)	1.73 (0.04)	0.99917
60	0.219 (0.123)	2.29 (0.05)	0.99637
90	0.353 (0.115)	2.95 (0.07)	0.99869
120	0.273 (0.101)	3.37 (0.04)	0.99775

^1^ Values in parentheses are the standard deviations of the data.

**Table 2:** Comparison of FF adsorption performances with other adsorbents.

Adsorbent	*q*_max_ (mg g^−1^)	Contact Time	pH	Isotherm Model	Reference
Coir pitch	4.31	24 h	3	Langmuir	[37]
Coconut shell	4.16	24 h	3	Langmuir	[37]
Durian peel	4.18	24 h	3	Langmuir	[37]
SB Functionalized fiber (FF)	4.86	1 h	5	Langmuir	This study
ACs from Rice Straw	181.19	1.5 h	2.35	Langmuir	[47]
ACs from Almond shell	188.90	—	2–8	Langmuir	[48]

## References

[1] US Environmental Protection Agency (USEPA) (2000). Environmental protection agency—Endocrine disruptor screening program. Report to Congress.

[2] Zeng G.M., Zhang C., Huang G.H., Yu J., Wang Q., Li J., Xi B., Liu H. (2006). Adsorption behavior of bisphenol A on sediments in Xiangjiang River, Central-south China. Chemosphere.

[3] Staples C.A., Dorn P.B., Klecka G.M., O’Block S.T., Harris L.R. (1998). A review of the environmental fate, effects, and exposures of bisphenol A. Chemosphere.

[4] Kang J.H., Kondo F., Katayama Y. (2006). Human exposure to bisphenol A. Toxicology.

[5] Huang Y.Q., Wong C.K.C., Zheng J.S., Bouwman H., Barra R., Wahlström B., Neretin L., Wong M.H. (2012). Bisphenol A (BPA) in China: A review of sources, environmental levels, and potential human health impacts. Environ. Int..

[6] Yamamoto T., Yasuhara A., Shiraishi H., Nakasugi O. (2001). Bisphenol A in hazardous waste landfill leachates. Chemosphere.

[7] Fukazawa H., Watanabe M., Shiraishi F., Shiraishi H., Shiozawa T., Matsushita H., Yoshiyasu T. (2002). Formation of chlorinated derivatives of Bisphenol A in waste paper recycling plants and their estrogenic activities. J. Health Sci..

[8] Heemken O.P., Reincke H., Stachel B., Theobald N. (2001). The occurrence of xenoestrogens in the Elbe river and the North Sea. Chemosphere.

[9] Lee H.B., Peart T.E., Gris G., Chan J. (2002). Endocrine-disrupting chemicals in industrial wastewater samples in Toronto, Ontario. Water Qual. Res. J. Can..

[10] Colin A., Bach C., Rosin C., Munoz J.F., Dauchy X. (2014). Is drinking water a major route of human exposure to alkylphenol and bisphenol contaminants in France?. Arch. Environ. Contam. Toxicol..

[11] Kleywegt S., Pileggi V., Yang P., Hao C., Zhao X., Rocks C., Thach S., Cheung P., Whitehead B. (2011). Pharmaceuticals, hormones and bisphenol A in untreated source and finished drinking water in Ontario, Canada, occurrence and treatment efficiency. Sci. Total Environ..

[12] Rochester J.R. (2013). Bisphenol A and human health: A review of the literature. Reprod. Toxicol..

[13] Castoria G., Giovannelli P., Lombardi M., De Rosa C., Giraldi T., de Falco A., Barone M.V., Abbondanza C., Migliaccio A., Auricchio F. (2012). Tyrosine phosphorylation of estradiol receptor by Src regulates its hormone-dependent nuclear export and cell cycle progression in breast cancer cells. Oncogene.

[14] Di Donato M., Cernera G., Giovannelli P., Galasso G., Bilancio A., Migliaccio A., Castoria G. (2017). Recent advances on bisphenol-A and endocrine disruptor effects on human prostate cancer. Mol. Cell. Endocrinol..

[15] Braun J.M., Kalkbrenner A.E., Calafat A.M., Yolton K., Ye X., Dietrich K.N., Lanphear B.P. (2011). Impact of early-life bisphenol A exposure on behavior and executive function in children. Pediatrics.

[16] Rees-Clayton E.M., Todd M., Dowd J.B., Aiello A.E. (2011). The impact of bisphenol A and triclosan on immune parameters in the U.S. population, NHANES 2003–2006. Environ. Health Persp..

[17] Joseph L., Flora J.R.V., Park Y.-G., Badawy M., Saleh H., Yoon Y. (2012). Removal of natural organic matter from potential drinking water sources by combined coagulation and adsorption using carbon nanomaterials. Sep. Purif. Technol..

[18] Westerhoff P., Yoon Y., Snyder S., Wert E. (2005). Fate of endocrine-disruptor, pharmaceutical, and personal care product chemicals during simulated drinking water treatment processes. Environ. Sci. Technol..

[19] Joseph L., Zaib Q., Khan I.A., Berge N.D., Park Y.-G., Saleh N.B., Yoon Y. (2011). Removal of bisphenol A and 17α-ethinyl estradiol from landfill leachate using single-walled carbon nanotubes. Water Res..

[20] Nam S.W., Jung C., Li H., Yu M., Flora J.R.V., Boateng L.K., Her N., Zoh K.D., Yoon Y. (2015). Adsorption characteristics of diclofenac and sulfamethoxazole to graphene oxide in aqueous solution. Chemosphere.

[21] Muhamad M.S., Salim M.R., Lau W.J., Yusop Z. (2016). A review on bisphenol A occurrences, health effects and treatment process via membrane technology for drinking water. Environ. Sci. Pollut. Res..

[22] de Oliveira L.K., Moura A.L.A., Barbosa V., Parreira R.L.T., Banegas R.S., Caramori G.F., Ciuffi K.J., Molina E.F. (2017). Removal of the emerging contaminant bisphenol A by an ureasil-PEO hybrid membrane: Experimental study and molecular dynamic simulation. Environ. Sci. Pollut. Res..

[23] Duan X.D., He X.X., Wang D., Mezyk S.P., Otto S.C., Marfil Vega R., Mills M.A., Dionysiou D.D. (2017). Decomposition of iodinated pharmaceuticals by UV254 nm-assisted advanced oxidation processes. J. Hazard. Mater..

[24] Wang L., Zhao J., Li Y. (2013). Removal of bisphenol A and 4-n-nonylphenol coupled to nitrate reduction using acclimated activated sludge under anaerobic conditions. J. Chem. Technol. Biotechnol..

[25] Wang G., Wu F., Zhang X., Luo M., Deng N. (2006). Enhanced photodegradation of bisphenol A in the presence of β-cyclodextrin under UV light. J. Chem. Technol. Biotechnol..

[26] Eio E.J., Kawai M., Niwa C., Ito M., Yamamoto S., Toda T. (2015). Biodegradation of bisphenol A by an algal-bacterial system. Environ. Sci. Pollut. Res..

[27] Davididou K., Hale E., Lane N., Chatzisymeon E., Pichavant A., Hochepied J.F. (2017). Photocatalytic treatment of saccharin and bisphenol-A in the presence of TiO_2_ nanocomposites tuned by Sn (IV). Catal. Today.

[28] Outsiou A., Frontistis Z., Ribeiro R.S., Antonopoulou M., Konstantinou I.K., Silva A.M.T., Faria J.L., Gomes H.T., Mantzavinos D. (2017). Activation of sodium persulfate by magnetic carbon xerogels (C_X_/CoFe) for the oxidation of bisphenol A: Process variables effects, matrix effects and reaction pathways. Water Res..

[29] Rivas F.J., Encinas A., Acedo B., Beltran F.J. (2008). Mineralization of bisphenol A by advanced oxidation processes. J. Chem. Technol. Biotechnol..

[30] Rivero M.J., Alonso E., Dominguez S., Ribao P., Ibañez R., Ortiz I., Irabien A. (2014). Kinetic analysis and biodegradability of the Fenton mineralization of bisphenol A. J. Chem. Technol. Biotechnol..

[31] Zacharakis A., Chatzisymeon E., Binas V., Frontistis Z., Venieri D., Mantzavinos D. (2013). Solar photocatalytic degradation of bisphenol A on immobilized ZnO or TiO_2_. Int. J. Photoenergy.

[32] Snyder S.A., Adham S., Redding A.M., Cannon F.S., DeCarolis J., Oppenheimer J., Wert E.C., Yoon Y. (2007). Role of membranes and activated carbon in the removal of endocrine disruptors and pharmaceuticals. Desalination.

[33] Ioannou-Ttofa L., Foteinis S., Chatzisymeon E., Fatta-Kassinos D. (2016). The environmental footprint of a membrane bioreactor treatment process through Life Cycle Analysis. Sci. Total Environ..

[34] Nakanishi A., Tamai M., Kawasaki N., Nakamura T., Tanada S. (2002). Adsorption characteristics of bisphenol A onto carbonaceous materials produced from wood chips as organic waste. J. Colloid Interface Sci..

[35] Asada T., Oikawa K., Kawata K., Ishihara S., Iyobe T. (2004). Study of removal effect of bisphenol-A and B-estradiol by porous carbon. J. Health Sci..

[36] Yoon Y., Westerhoff P., Snyder S.A., Esparza M. (2003). HPLC fluorescence detection and adsorption of bisphenol A, 17β-estradiol, and 17α-ethynyl estradiol on powdered activated carbon. Water Res..

[37] Lazim Z.M., Hadibarata T., Puteh M.H., Yusop Z. (2015). Adsorption characteristics of bisphenol A onto low-cost modified phyto-waste material in aqueous solution. Water Air Soil Pollut..

[38] Tursi A., Beneduci A., Chidichimo F., De Vietro N., Chidichimo G. (2018). Remediation of hydrocarbons polluted water by hydrophobic functionalized cellulose. Chemosphere.

[39] Arias F., Beneduci A., Chidichimo F., Furia E., Straface S. (2017). Study of the adsorption of mercury (II) on lignocellulosic materials under static and dynamic conditions. Chemosphere.

[40] Gabriele B., Cerchiara T., Salerno G., Chidichimo G., Vetere M.V., Alampi C., Gallucci M.C., Conidi C., Cassano A. (2009). A new physical-chemical process for the efficient production of cellulose fibers from Spanish broom (Spartium junceum L.). Bioresour. Technol..

[41] Agenzia Nazionale per la protezione dell’Ambiente (APAT) (2003). Metodi analitici per le acque/APAT; IRSA-CNR. Manuali e Linee Guida 29/2003.

[42] Henze M., Comeau Y., Henze M., van Loosdrecht M.C.M., Ekama G.A., Brdjanovic D. (2008). Wastewater characterization. Biological Wastewater Treatment: Principles, Modelling and Design.

[43] Boehm H.P., Bottani E.J., Tascón J.M.D. (2008). Chapter thirteen—Surface chemical characterization of carbons from adsorption studies. Adsorption by Carbons.

[44] Bismarck A., Aranberri-Askargorta I., Springer J., Lampke T., Wielage B., Stamboulis A., Limbach H.H. (2002). Surface characterization of flax, hemp and cellulose fibers; surface properties and the water uptake behavior. Polym. Compos..

[45] Limousin G., Gaudet J.P., Charlet L., Szenknect S., Barthes V., Krimissa M. (2007). Sorption isotherms: A review on physical bases, modeling and measurement. Appl. Geochem..

[46] Atkins P., De Paula J. (2006). Physical Chemistry.

[47] Chang K.L., Hsieh J.F., Ou B.M., Chang M.H., Hseih W.Y., Lin J.H., Huang P.J., Wong K.F., Chen S.T. (2012). Adsorption studies on the removal of an endocrine-disrupting compound (bisphenol A) using activated carbon from rice straw agricultural waste. Sep. Sci. Technol..

[48] Bautista-Toledo I., Ferro-Garcia M.A., Rivera-Utrilla J., Moreno-Castilla C., Vegas Fernandez F.J. (2005). Bisphenol A removal from water by activated carbon. Effect of carbon characteristics and solution chemistry. Environ. Sci. Technol..

[49] Cerchiara T., Chidichimo G., Rondi G., Gallucci M.C., Gattuso C., Luppi B., Bigucci F. (2014). Chemical Composition, morphology and tensile properties of Spanish broom (Spartium junceum L.) fibres in comparison with flax (Linum usitatissimum L.). Fibres Text. East. Eur..

